# Role of heme in lung bacterial infection after trauma hemorrhage and stored red blood cell transfusion: A preclinical experimental study

**DOI:** 10.1371/journal.pmed.1002522

**Published:** 2018-03-09

**Authors:** Brant M. Wagener, Parker J. Hu, Joo-Yeun Oh, Cilina A. Evans, Jillian R. Richter, Jaideep Honavar, Angela P. Brandon, Judy Creighton, Shannon W. Stephens, Charity Morgan, Randal O. Dull, Marisa B. Marques, Jeffrey D. Kerby, Jean-Francois Pittet, Rakesh P. Patel

**Affiliations:** 1 Department of Anesthesiology and Perioperative Medicine, University of Alabama at Birmingham, Birmingham, Alabama, United States of America; 2 Department of Surgery, University of Alabama at Birmingham, Birmingham, Alabama, United States of America; 3 Department of Pathology, University of Alabama at Birmingham, Birmingham, Alabama, United States of America; 4 Department of Emergency Medicine, University of Alabama at Birmingham, Birmingham, Alabama, United States of America; 5 Department of Biostatistics, University of Alabama at Birmingham, Birmingham, Alabama, United States of America; 6 Department of Anesthesiology, University of Illinois at Chicago, Chicago, Illinois, United States of America; 7 Center for Free Radical Biology, University of Alabama at Birmingham, Birmingham, Alabama, United States of America; Barts and the London School of Medicine & Dentistry Queen Mary University of London, UNITED KINGDOM

## Abstract

**Background:**

Trauma is the leading cause of death and disability in patients aged 1–46 y. Severely injured patients experience considerable blood loss and hemorrhagic shock requiring treatment with massive transfusion of red blood cells (RBCs). Preclinical and retrospective human studies in trauma patients have suggested that poorer therapeutic efficacy, increased severity of organ injury, and increased bacterial infection are associated with transfusion of large volumes of stored RBCs, although the mechanisms are not fully understood.

**Methods and findings:**

We developed a murine model of trauma hemorrhage (TH) followed by resuscitation with plasma and leukoreduced RBCs (in a 1:1 ratio) that were banked for 0 (fresh) or 14 (stored) days. Two days later, lungs were infected with *Pseudomonas aeruginosa* K-strain (PAK). Resuscitation with stored RBCs significantly increased the severity of lung injury caused by *P*. *aeruginosa*, as demonstrated by higher mortality (median survival 35 h for fresh RBC group and 8 h for stored RBC group; *p <* 0.001), increased pulmonary edema (mean [95% CI] 106.4 μl [88.5–124.3] for fresh RBCs and 192.5 μl [140.9–244.0] for stored RBCs; *p =* 0.003), and higher bacterial numbers in the lung (mean [95% CI] 1.2 × 10^7^ [−1.0 × 10^7^ to 2.5 × 10^7^] for fresh RBCs and 3.6 × 10^7^ [2.5 × 10^7^ to 4.7 × 10^7^] for stored RBCs; *p =* 0.014). The mechanism underlying this increased infection susceptibility and severity was free-heme-dependent, as recombinant hemopexin or pharmacological inhibition or genetic deletion of *toll-like receptor 4 (TLR4)* during TH and resuscitation completely prevented *P*. *aeruginosa–*induced mortality after stored RBC transfusion (*p* < 0.001 for all groups relative to stored RBC group). Evidence from studies transfusing fresh and stored RBCs mixed with stored and fresh RBC supernatants, respectively, indicated that heme arising both during storage and from RBC hemolysis post-resuscitation plays a role in increased mortality after PAK (*p* < 0.001). Heme also increased endothelial permeability and inhibited macrophage-dependent phagocytosis in cultured cells. Stored RBCs also increased circulating high mobility group box 1 (HMGB1; mean [95% CI] 15.4 ng/ml [6.7–24.0] for fresh RBCs and 50.3 ng/ml [12.3–88.2] for stored RBCs), and anti-HMGB1 blocking antibody protected against PAK-induced mortality in vivo (*p =* 0.001) and restored macrophage-dependent phagocytosis of *P*. *aeruginosa* in vitro. Finally, we showed that TH patients, admitted to the University of Alabama at Birmingham ER between 1 January 2015 and 30 April 2016 (*n =* 50), received high micromolar–millimolar levels of heme proportional to the number of units transfused, sufficient to overwhelm endogenous hemopexin levels early after TH and resuscitation. Limitations of the study include lack of assessment of temporal changes in different products of hemolysis after resuscitation and the small sample size precluding testing of associations between heme levels and adverse outcomes in resuscitated TH patients.

**Conclusions:**

We provide evidence that large volume resuscitation with stored blood, compared to fresh blood, in mice increases mortality from subsequent pneumonia, which occurs via mechanisms sensitive to hemopexin and TLR4 and HMGB1 inhibition.

## Abstract 2

Rakesh Patel and colleagues use a mouse model of trauma and hemorrhagic shock to investigate mechanisms underlying clinically observed associations between stored versus fresh red blood cell transfusion, and bacterial infection or organ injury.

## Author summary

**Why was this study done?:**

- Trauma hemorrhage patients receive high-volume resuscitations of stored red blood cells (RBCs), but whether transfusion with older, compared to fresher, stored RBCs is safe remains unclear.
- Retrospective analyses of clinical data indicate that adverse effects associated with transfusion of older stored RBCs to trauma hemorrhage patients include increased risk for nosocomial infections and mortality, but potential mechanisms underlying these associations are poorly understood.
- Our goal was to determine the mechanistic basis linking older stored RBC transfusions and bacterial pneumonia after hemorrhagic shock, which, if elucidated, may reveal new approaches to improve the safety of stored RBC transfusions.

**What did the researchers do and find?:**

- We used a murine model of trauma hemorrhage with transfusion of fresh or stored mouse RBCs, followed by airway instillation of *Pseudomonas aeruginosa* 2 days after resuscitation; indices pertinent to assessing severity of bacterial pneumonia were measured.
- We found that mice that received stored RBCs were significantly more susceptible to *P*. *aeruginosa*–induced lung leak and death compared to mice that received fresh RBCs, with a central role for free heme suggested by the protection afforded by administration of hemopexin at the time of resuscitation.
- Using in vivo and cell-culture studies, we found that free heme mediates the increased severity of bacterial pneumonia after trauma, hemorrhage, and resuscitation with stored RBCs, via TLR4- and HMGB1-dependent mechanisms of increased endothelial permeability and decreased macrophage-dependent bacterial clearance.

**What do these findings mean?:**

- These data suggest that free heme is a key player in adverse effects associated with stored RBC transfusion after trauma hemorrhage.
- These data suggest strategies to limit heme exposure or enhance endogenous mechanisms to prevent heme toxicity, which may improve the safety of stored RBC transfusions.

## Introduction

Transfusion with stored red blood cells (RBCs) is an integral component of resuscitation strategies for a number of clinical scenarios. Currently in the United States, RBCs stored for up to 42 d are approved for transfusion. However, several recent studies have raised questions surrounding whether, even for RBCs below the 42-d threshold, transfusion with older compared to younger RBCs is therapeutically efficacious and, more importantly, safe [1]. Specifically, the majority of these analyses report that transfusion of older compared to younger RBCs is associated with adverse effects [2–12] in acute critical illness and surgery. In contrast, recent data from prospective clinical studies indicate that there is no difference in outcome between transfusion with younger and older RBCs in cardiac surgery and critically ill patients [13–16]. However, in these studies, patients were not receiving blood transfusions for hemorrhagic shock, and “older” RBCs were those stored for 20–26 d, precluding assessment of potential effects of RBCs stored for longer periods. This is important as many of the changes in stored RBCs, including in-bag hemolysis and extravascular hemolysis (monocyte/macrophage-dependent clearance of RBCs) post-transfusion increase significantly during the last week of storage [17–19]. Additionally, recent retrospective analyses of clinical data indicate adverse effects in high-risk patients with RBCs stored for >35 d [20]. Moreover, most studies do not address any RBC dose–response effects that would normally be associated with patients receiving massive transfusion. Our previous studies have shown increased risk for adverse effects in trauma hemorrhage (TH) patients as a function of both RBC storage age and the number of RBCs transfused [21].

TH is the major cause of morbidity and mortality in patients aged 1–46 y. Severely traumatized patients are often in hemorrhagic shock and thus require massive blood transfusion. These patients may receive the oldest RBCs available in order to avoid wastage. Our previous studies have shown that the ability of transfused RBCs to improve microcirculatory function and tissue oxygenation in trauma patients progressively diminishes with RBC storage age [22]. Storage of RBCs is accompanied by biochemical and morphological changes referred to as “storage lesion.” Key amongst these changes are hemolysis and release of free and microvesicle-associated hemoglobin, free heme, and iron [23–25]. Transfusion of these molecular species increases oxidative stress, inhibits nitric oxide bioavailability, results in a pro-inflammatory state, and provides substrate (low molecular weight iron) for microbial growth [17,18,26–39]. Additionally, a recent review showed that of 15 clinical retrospective studies evaluating RBC transfusion in trauma patients between 1999 and 2013, all but 1 reported adverse effects of older stored RBCs [40]. Retrospective analyses also demonstrate positive associations between the storage age and number of RBC units transfused and increased end organ injury, nosocomial infections, and mortality [11,21,22,41–43].

In this study, we explored the mechanisms underlying increased risk for nosocomial infection in the setting of TH and massive transfusion with younger versus older stored RBCs and specifically tested the hypothesis that free heme, which has emerged as a key mediator of pro-inflammatory and pro-oxidative stress reactions in critical illnesses [7,36,44–47], plays an important role in this increased risk for infection. We used a murine model of TH with transfusion of fresh or stored mouse RBCs, followed by airway instillation of *Pseudomonas aeruginosa* and assessment of the severity of bacterial pneumonia. Complementary cell-culture studies assessing the effects and mechanisms of heme-dependent inhibition of bacterial killing by immune cells and observational studies measuring products of hemolysis in transfused TH patients were also performed.

## Methods

### Ethic statement

All research involving human participants was approved by the University of Alabama at Birmingham (UAB) Institutional Review Board.

### Reagents

All reagents were obtained from Fisher Scientific (Hampton, NH) unless otherwise specified. TAK-242 was purchased from InvivoGen (San Diego, CA). Hemopexin (HPX) was purchased from Athens Research and Technology (Athens, GA). Anti-HMGB1 blocking antibody was a kind gift from Kevin Tracey (Feinstein Institute for Medical Research, New York, NY). Intralipid, IgG2b isotype blocking antibody, iron (III) chloride, neocuproine hydrochloride, ferrozine, ascorbic acid, and nitrilotriacetic acid (NTA) were purchased from Sigma-Aldrich (Saint Louis, MO). Heme (hemin chloride) was purchased from Frontier Scientific (Logan, UT) and prepared fresh on the day of each experiment in 0.1 M NaOH, then diluted to 100 μM in PBS (pH 7.4).

### Mice

C57BL/6 mice were purchased from Charles River Laboratories (Wilmington, MA) for all experiments. Mice were housed in UAB animal care facilities under 12-h light/dark cycles and received lab diet NIH-31 (PMI Nutrition International, Brentwood, MO) ad libitum. Mice were acclimated for at least 5 d prior to initiation of experiments and had access to water and food (diet NIH-31 TAC auto/irradiated, PMI Nutrition International) ad libitum. All animal experiments were carried out according to protocols approved by the UAB Institutional Animal Care and Use Committee and were conducted according to the Guide for the Care and Use of Laboratory Animals [48].

### Rat endothelial cell isolation

Rat microvascular endothelial cells (RMVECs) were isolated and cultured as previously described [49]. Briefly, rats were euthanized and underwent sternotomy, and heart and lungs were excised en bloc. For RMVEC isolation, thin strips removed from the lung parenchyma were minced and digested. Complete medium as described below was added to the isolation mixture, and cells were centrifuged, resuspended, and plated in culture dishes. RMVECs were removed using trypsin and cloning rings and resuspended in complete medium. Primary cultures were characterized by their cobblestone morphology, uptake of 1,1′-dioctadecyl-3,3,3′,3′-tetramethylindocarbocyanine-labeled low-density lipoprotein, and an endothelial cell marker panel.

### Cell culture

RMVECs were cultured in Dulbecco’s Modified Eagle Medium, and a murine alveolar macrophage cell line (MH-S cells, no. CRL-2019; American Type Culture Collection, Manassas, VA) was cultured in Roswell Park Memorial Institute 1640 Medium. Both were supplemented with 10% fetal bovine serum and 1% penicillin/streptomycin and were maintained at 37°C with 5% CO_2_.

### Mouse blood banking

Mouse blood banking was performed as previously described with slight modifications [36,39]. Blood from C57BL/6 male mice was collected by cardiac puncture and leukoreduced under sterile conditions by filtering through a Sephadex G25 microcellulose column (1:3 w/w). Columns were then washed with 10 times the volume of PBS. Preliminary studies demonstrated >99% loss of leukocytes and 95% ± 2.4% loss of platelets (mean ± SEM, *n =* 3) by FACS analyses. Briefly, 250 μl of whole blood was sampled before and after leukoreduction, acting as its own control. Anti-CD41 was added to a final concentration of 1:500. Collected RBCs were centrifuged at 3,000 × *g* for 5 min at 4°C, concentrated to a hematocrit of 60% with AS-1 (Baxter, Lake Zurich, IL), and then placed in a capped 1-ml syringe with no headspace. RBCs (0.4 ml total volume at 60% Hct) were stored at 4°C for up to 14 d in the dark. Storage of murine RBCs for approximately 2 wk is equivalent to human RBC storage for 42 d based on RBC stability and recovery post-transfusion [29,34,36,50]. In separate experiments, fresh and stored RBCs and supernatants were separated by centrifugation (3,000 × *g* for 5 min at 4°C). Then, the RBC fraction from a fresh sample was mixed with supernatants from a stored sample, and the RBC fraction from a stored sample mixed with supernatants from a fresh sample. Each mixture was then used immediately in resuscitation.

### Plasma collection and storage

Frozen C57BL/6 mouse plasma was purchased from Innovative Research (Novi, MI) and stored at −20°C in 100-μl aliquots. On the day of each experiment, plasma was thawed, mixed with stored RBCs, and then transfused.

### TH resuscitation model

The TH resuscitation mouse model was performed as previously described [36]. All surgeries were initiated between 7 AM and 9 AM. C57BL/6 male mice (22–26 g) were anesthetized by inhalation of 5% isoflurane in air. The concentration of isoflurane was then reduced to the minimal concentration for maintenance. The abdomen and groin were shaved and washed with 10% povidone-iodine, and a 2-cm midline laparotomy was performed to induce soft tissue trauma. Both femoral arteries were cannulated with catheters (Braintree Scientific, Braintree, MA). Systemic arterial pressure was continuously monitored through one arterial line while hemorrhage and resuscitation were performed via the other. Mice were bled over 30 min to a mean arterial pressure of 30 ± 5 mm Hg. This blood pressure was maintained for a further 30 min by additional bleeding as required. All incision sites were bathed with 2% lidocaine for analgesia and re-bathed as needed. At the end of the 60-min hemorrhagic shock period, animals were resuscitated over 30 min with 3 units (300 μl) of fresh (day 0, *n =* 8) or stored (day 14, *n =* 9) packed RBCs. Given recent results from the PROPPR trial indicating that initial transfusion of packed RBCs and plasma should occur in a 1:1 ratio [51], we resuscitated our hemorrhaged mice with equivalent volumes of RBCs and plasma. After resuscitation, mice were placed into cages with a heating pad for approximately 1 h and then moved to standard housing. When applicable, TAK-242 (7.5 mg/kg) was administered intravenously immediately after the onset of shock to allow comparison and validation of data collected with *TLR4*^−/−^ mice. Hemopexin (1 mg/kg) was administered intravenously before the onset of resuscitation, immediately prior to when heme exposure via stored RBC transfusion occurred. Anti-HMGB1 blocking antibody (50 μg) was administered intraperitoneally before tracheal instillation of *P*. *aeruginosa*.

### Measurement of hemoglobin, free heme, and non-transferrin-bound iron

At indicated times, blood was collected from the mice and immediately centrifuged (2,000 × g, 2 min, room temperature) to pellet RBCs. Plasma was separated, stored on ice and free hemoglobin and heme measured within 1 h of collection by spectral deconvolution as recently described [52]. Similar methods were used to assess free hemoglobin and cell-free heme (CFH) in stored RBCs. Non-transferrin-bound iron (NTBI) was measured with slight modifications as described previously [53–55]. Briefly, 152 μl of iron (III) chloride standard (0, 0.1, 0.5, 1, 5, 10, and 50 μM) or mice plasma was incubated with 8 μl of 1.6 M NTA (pH 7.0, final concentration 80 mM) for 30 min at 20–25°C. Then 160 μl of standard or plasma sample in 80 mM NTA was ultrafiltered using Centricon (3 kDa MW; Millipore, Billerica, MA) at 10,620 × *g* (10,000 rpm) for 2 h at 15°C. Then 120 μl of eluted standard or plasma sample was acidified with 6.3 μl of 100 mM HCl (final concentration 5 mM) in 96-well plates and incubated for 30 min at room temperature. Then 44 μl of the iron-detection reagent (final concentration 0.5 mM ferrozine, 0.5 mM neocuproine, 0.21 M ammonium acetate, and 0.07 M ascorbic acid in water) was added to the wells and incubated for 1 h. Ferrous ferrozine complex was measured at 540 nm by VICTOR plate reader (PerkinElmer, Waltham, MA), and plasma NTBI was calculated according to iron (III) chloride standard curve. The concentration of standard was verified by extinction coefficient of ferrous ferrozine complex at 562 nm (27.9 mM^−1^cm^−1^).

### Pneumonia model

The mouse pneumonia model was performed as we previously described [56]. Mice were instilled with *P*. *aeruginosa* K-strain (PAK), a wild-type strain (a gift from Dr. Stephen Lory), 48 h after TH and resuscitation. In separate studies, PAK was administered to wild-type mice without TH and resuscitation and immediately after TAK-242 or hemopexin administration or 48 h post-hemopexin administration. Briefly, mice were anesthetized with ketamine/xylazine (90–200 mg/kg/10 mg/kg intramuscularly). The mouse was laid on a board with its head elevated at 45°, and 25 μl of phosphate-buffered saline containing approximately 5 × 10^7^ colony-forming units (CFUs) of *P*. *aeruginosa* was instilled into both lungs through the trachea via the mouth by using a 27G gavage needle. The mouse was allowed to recover for 15 min prior to being returned to the cage. Mice were active and appeared normal after 30 min. When applicable, 6 h after the bacterial instillation, mice were euthanized with a larger dose of ketamine/xylazine. Blood samples were collected in a sterile fashion through puncture of the inferior vena cava after laparotomy and bilateral thoracotomies were performed. The mouse lungs were removed, weighed, and homogenized for lung vascular permeability or bacterial CFU measurements.

### Survival

Following procedures, mice were weighed twice a day and carefully monitored every 6 h to follow the anticipated development of a systemic inflammatory response secondary to the shock period. This response was graded using a clinical index of systemic inflammation based on the presence of the following signs: lethargy, piloerection, tremors, periorbital exudates, labored respirations, and diarrhea. Mice displaying signs of overt distress or respiratory distress (coughing, gasping, insufficient gas exchange leading to hypoxia) or clearly moribund were euthanized. Possible hind-limb ischemia secondary to the femoral artery ligations was also monitored by examination for lack of perfusion (pink coloration and cold) and, if observed, triggered euthanasia. Euthanasia was carried out within 15 min upon reaching end point criteria. After PAK instillation, mice were checked until death or 96 h, except for experiments testing the effects of anti-HMGB1 blocking antibody, where survival was monitored only for 24 h after PAK instillation. Survival time was defined as the time between instillation and death. Mice displaying signs of respiratory distress (coughing, gasping, insufficient gas exchange leading to hypoxia) secondary to multi-organ dysfunction were euthanized, and time of death recorded. Therefore, survival end points reflected a composite of mortality and respiratory distress secondary to multi-organ dysfunction. To avoid unnecessary duplication and use of mice, survival data are pooled from multiple experiments conducted over 18 mo, for a total of 42 mice. Effects of fresh and stored RBCs on this end point were assessed over this entire period to evaluate consistency of responses across time and to allow for comparison of therapeutic (hemopexin, TAK-242) and TLR4 depletion effects.

### Lung vascular permeability measurement

Lung endothelial permeability to protein (percent) and excess lung water (microliters) were measured as previously described and plotted as extravascular pulmonary equivalents (EVPE) [56]. Briefly, 0.5 μCi of ^125^I-albumin was injected intravenously; blood collected through puncture of the inferior vena cava; lungs removed, weighed, and homogenized; and radioactivity counted in a Wizard γ-counter (PerkinElmer). The homogenate was weighed and a fraction centrifuged (12,000 × *g*, 8 min) to assay for hemoglobin concentration in the supernatant. Endothelial permeability was calculated as the counts of ^125^I-albumin in the blood-free lung tissue divided by the counts of ^125^I-albumin in the plasma. For these studies, mice were sacrificed 6 h after PAK instillation and represent a separate group from animals used to assess survival or bacterial CFUs.

### Measurement of bacterial CFUs

Lungs were collected in a sterile fashion as previously described [56]. The lungs were homogenized in sterile containers, and the homogenates were serially diluted and plated in triplicate on agar plates. For these studies, mice were sacrificed 6 h after PAK instillation and represent a separate group from animals used to assess survival or permeability.

### Transendothelial permeability

Endothelial barrier integrity was measured using an Electric Cell-substrate Impedance Sensing (ECIS) system (Applied BioPhysics, Troy, NY) as previously described [49]. Briefly, RMVECs were plated onto 8W10E+ arrays in normal culture medium and used when resistance reached ±900 Ohm, usually 24 h after seeding. Resistance was measured every 10 min for the duration of the experiments. Baseline resistance was measured for 1 h before addition of TAK-242, hemopexin, or PAK. TAK-242 and hemopexin were added 30 min before PAK.

### HMGB1 measurement

Confluent RMVECs were exposed to 10 μM hemin for 6 h, and supernatants were collected and stored at −80°C until assayed. HMGB1 levels were measured by ELISA according to manufacturer instructions (IBL International, Morrisville, NC).

### Phagocytosis

The assay was performed as we have previously described [56]. Briefly, 1 × 10^6^ MH-S cells, a murine alveolar macrophage cell line, were exposed to 10^7^ CFU/ml of PAK for 45 min at 37°C. Gentamicin (150 μg/ml) was added, and cells were incubated for 1 h at 37°C. The medium was removed, and cells were washed twice with sterile PBS then lysed by adding 200 μl of hypotonic buffer (pH 7.2) and incubated on ice for 10 min. Sterile water (800 μl) was added to the cell suspension, which was serially diluted onto agar plates. The plates were incubated for 24 h at 37°C, and the resulting colonies counted. No bacterial colonies were observed in lysates from uninfected macrophages or when bacteria alone were incubated for 1 h with gentamicin, indicating complete killing using this protocol.

### TH patients

One hundred consecutive trauma patients admitted to the UAB ER from 1 January 2015 to 30 April 2016 were screened by the attending trauma surgeon for inclusion into the study. Inclusion criteria were blunt or penetrating trauma, level I trauma activation, and systolic blood pressure < 90 mm Hg, respiratory compromise or placement of an advanced airway, or a Glasgow Coma Score < 9. Patients were excluded if they were <19 y old; consent was denied, not returned, or not obtainable due to no family available for consenting (due to the nature of the study, investigators had 72 h from the time of admission to obtain informed consent from the patient or an appropriate surrogate); or patients who had a bleeding diathesis or were known to take anticoagulant medications, had known liver disease, were pregnant, were incarcerated, or expired within 1 h of admission (*n =* 44). A blood sample was drawn at the conclusion of damage control surgery and blood product resuscitation after both the surgeon and anesthesiologist (where applicable) agreed that hemodynamic resuscitation was complete, an approach implemented in the PROPPR study, for which the UAB ER was one of the recruiting centers [51]. Generally, damage control surgery and resuscitation took no longer than 3 h to complete. An additional 6 individuals were excluded due to incomplete sample collection or processing. RBCs were pelleted by centrifugation at 2,000 × *g* for 2 min, and cell-free Hb, free heme, and NTBI in the plasma measured as described above. Also, segments were collected from all blood bags transfused, and data analyzed for 35 patients for whom segments from each unit transfused were available. RBCs were collected from segments and pelleted by centrifugation (3,000 × *g* for 5 min at 4°C). Supernatants were collected, and concentrations of oxyhemoglobin (oxyHb), methemoglobin (metHb), and free heme determined as outlined above. All research involving human participants was approved by the UAB Institutional Review Board. Whole blood was collected via a central line in acid citrate dextrose vacutainers and centrifuged within 60 min of collection. Plasma was separated and stored at 4°C for up to 72 h before being rapidly frozen in liquid nitrogen and stored at −80°C for further analysis. Transfused RBCs included in these analyses were predominantly stored in AS-1 (87%), with the remainder in AS-3 (7%) or CPDA-1 (6%) storage solution.

### Plasma haptoglobin and hemopexin

Human haptoglobin and hemopexin were determined by ELISA sandwich assay (ab108858 and ab171576 for human; Abcam, Cambridge, MA) according to the manufacturer instructions.

### Statistical analysis

All normally distributed data were summarized as mean ± SEM. Non-normally distributed data were summarized as median ± interquartile range. For the statistical analysis, we used GraphPad Prism (GraphPad Software, La Jolla, CA). Normality was assessed using the D’Agostino–Pearson test and through visual inspections of probability plots. For normally distributed data, 1-way ANOVA followed by a Tukey post-test was used to compare 3 or more experimental groups; for non-normally distributed data, the Kruskal–Wallis test followed by Dunn’s multiple comparison test was used. Student *t* test was used to compare 2 experimental groups. A Kaplan–Meier analysis followed by a log-rank (Mantel–Cox) test was used to compare the survival between 2 experimental groups. A *p*-value of <0.05 was considered statistically significant.

## Results

### TH resuscitation with stored blood increases mortality, pulmonary edema formation, and bacterial infection from subsequent *P*. *aeruginosa* pneumonia

**Fig 1:**
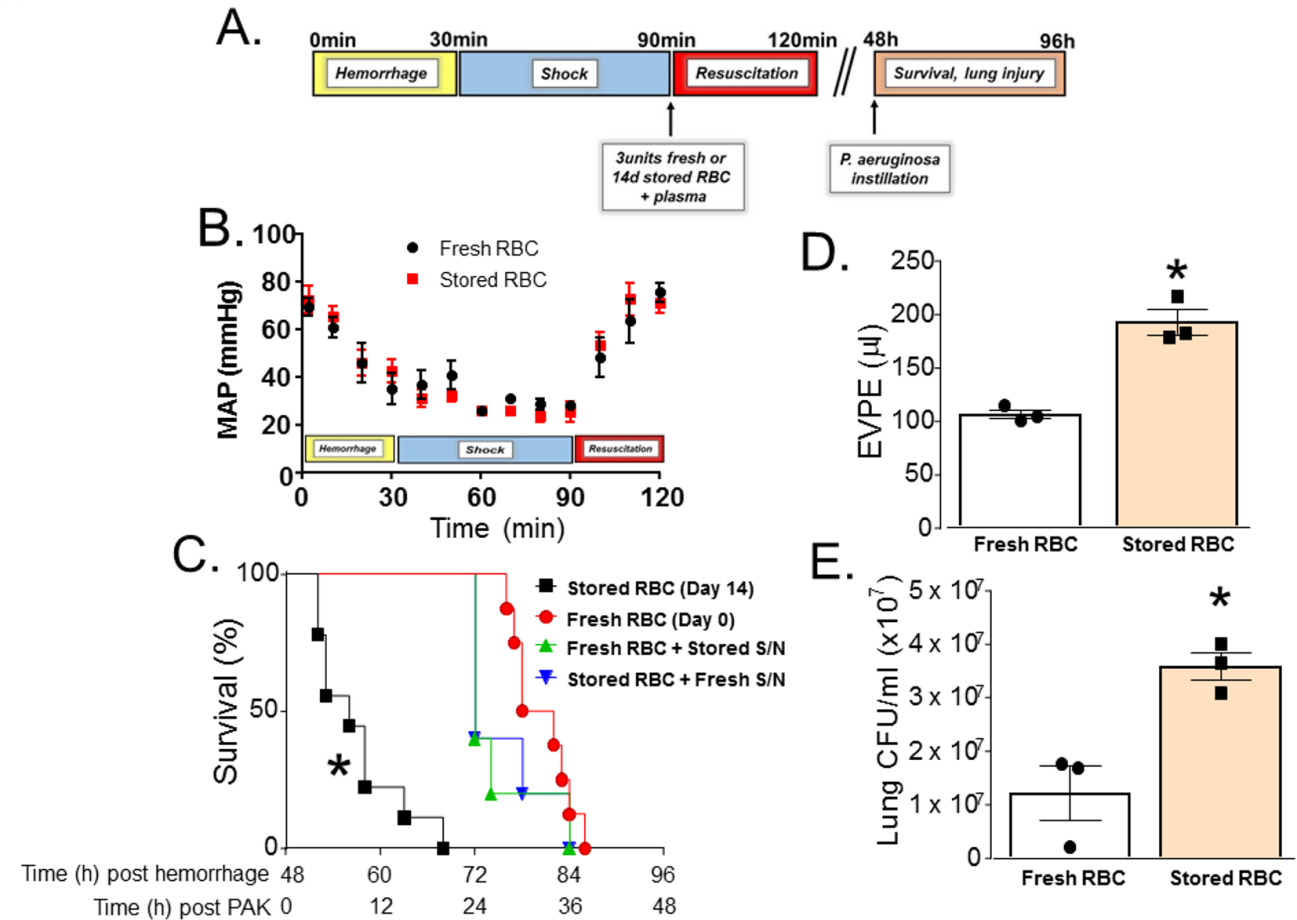
Trauma hemorrhage (TH) and resuscitation with stored blood increases mortality, pulmonary edema, and bacterial colony-forming units (CFUs) induced by subsequent *P*. *aeruginosa* pneumonia. (A) Scheme of experimental protocol used. (B) Mean arterial pressure (MAP) readings during hemorrhage, shock, and resuscitation with 3 units of fresh (day 0, *n =* 8) or stored (14 d, *n =* 8) red blood cells (RBCs). Data shown are mean ± SEM. (C) C57BL/6 wild-type mice underwent TH and were randomly resuscitated with either fresh (0 storage days, circles, *n =* 12) or stored (14 storage days, squares, *n =* 9) RBCs with plasma (1:1). Forty-eight hours later, these mice were instilled with *P*. *aeruginosa* K-strain (PAK), and survival monitored. *p < 0.001 compared to fresh RBC resuscitation by log-rank test. Also shown are effects of PAK after mice were transfused with fresh packed RBCs mixed with stored supernatants (S/N, triangles, *n =* 5, *p <* 0.001) or stored packed RBCs mixed with fresh supernatants (inverted triangles, *n =* 5, *p <* 0.001); *p-*values indicate comparison to stored RBC resuscitation group by log-rank test. (D) Mice underwent TH and resuscitation with either fresh (*n =* 3) or stored (*n =* 3) RBCs and 6 h after PAK lung instillation were sacrificed and extravascular pulmonary equivalents (EVPE) measured. Data are mean ± SEM; each data point represents a separate animal. *p = 0.003 by unpaired *t* test. (E) Mice underwent TH and resuscitation with either fresh (*n =* 3) or stored (*n =* 3) RBCs and 6 h after PAK instillation were sacrificed and lung CFU/ml was measured according to protocol. Data are mean ± SEM; each data point represents a separate animal. *p = 0.014 by unpaired *t* test.

C57BL/6 mice underwent TH and resuscitation with fresh or stored RBCs and plasma (Fig 1A). No differences in blood pressure changes before or after resuscitation between groups were observed, consistent with equal volume transfusions (Fig 1B). Two days after resuscitation, mice had their trachea instilled with PAK (wild-type *P*. *aeruginosa*); Fig 1A illustrates the experimental protocol. Median survival was 32 h for mice transfused with fresh RBCs (*n =* 12). However, mortality significantly increased in mice resuscitated with stored RBCs (median survival 8 h, *n =* 9), with all mice dying within 20 h of PAK instillation (Fig 1C). In a separate group of mice, pulmonary edema (*n =* 3) and bacterial CFUs (*n =* 3) were measured. Pulmonary edema approximately doubled (Fig 1D), and lung bacterial CFUs significantly increased (Fig 1E) in mice resuscitated with stored RBCs compared to those that received fresh RBCs. These results indicate that in the setting of trauma with hemorrhagic shock, massive resuscitation with stored RBCs may significantly impair bacterial clearance in the lung compared to resuscitation with fresh RBCs. We also tested whether the effects of stored RBCs were due to RBCs and/or supernatants in stored samples. Pelleted RBCs from 14-d stored RBCs were mixed with supernatants from a fresh RBC (0 d) preparation and vice versa. Mice underwent TH and resuscitation with these mixtures, and 48 h later PAK was instilled and survival assessed. Fig 1C shows that mice resuscitated with fresh RBCs plus stored supernatants (*n =* 5) or stored RBCs plus fresh supernatants (*n =* 5) had improved survival (median survival 24 h) compared to the stored RBC group.

### Impairment of lung function secondary to TH and massive resuscitation with stored RBCs and subsequent pneumonia is heme-dependent

**Fig 2:**
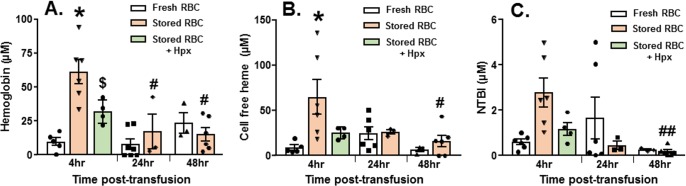
Changes in plasma hemoglobin, heme, and non-transferrin-bound iron (NTBI) after trauma hemorrhage (TH) and resuscitation with stored blood. At various times after TH and resuscitation with fresh (day 0) or stored (day 14) red blood cells (RBCs), mouse plasma was collected and free hemoglobin (A), heme (B), and NTBI (C) measured. Each symbol represents an individual sample. Data are mean ± SEM; *p < 0.001 relative to fresh RBCs at 4 h. ^#^*p <* 0.001 and ^##^*p =* 0.02 relative to stored RBCs at 4 h by 2-way ANOVA with Tukey post-test. ^$^*p =* 0.034 by unpaired *t* test relative to stored RBCs at 4 h.

There are numerous components in stored RBCs that have the potential to cause organ injury that include, but are not limited to, free heme, free hemoglobin, iron, and microvesicles [25,52]. Consistent with our previous studies [25,36], free oxyHb, metHb, and heme levels were increased after 14 d of RBC storage, being 930 ± 125 μM, 40.3 ± 8.8 μM, and 168 ± 44.7 μM, respectively (mean ± SEM, *n =* 7). Fig 2 shows that free hemoglobin and free heme were higher 4 h after resuscitation with stored RBCs compared to fresh RBCs (*n =* 3–7 as indicated); NTBI level was also higher, but this difference did not reach statistical significance (*p =* 0.07). Levels of these mediators decreased after 24–48 h. Based on our previous studies and studies from other groups indicating a role of free heme in mediating inflammatory end organ injury [36,57] after transfusion with stored RBCs, we next tested the effects of exogenous hemopexin.

**Fig 3:**
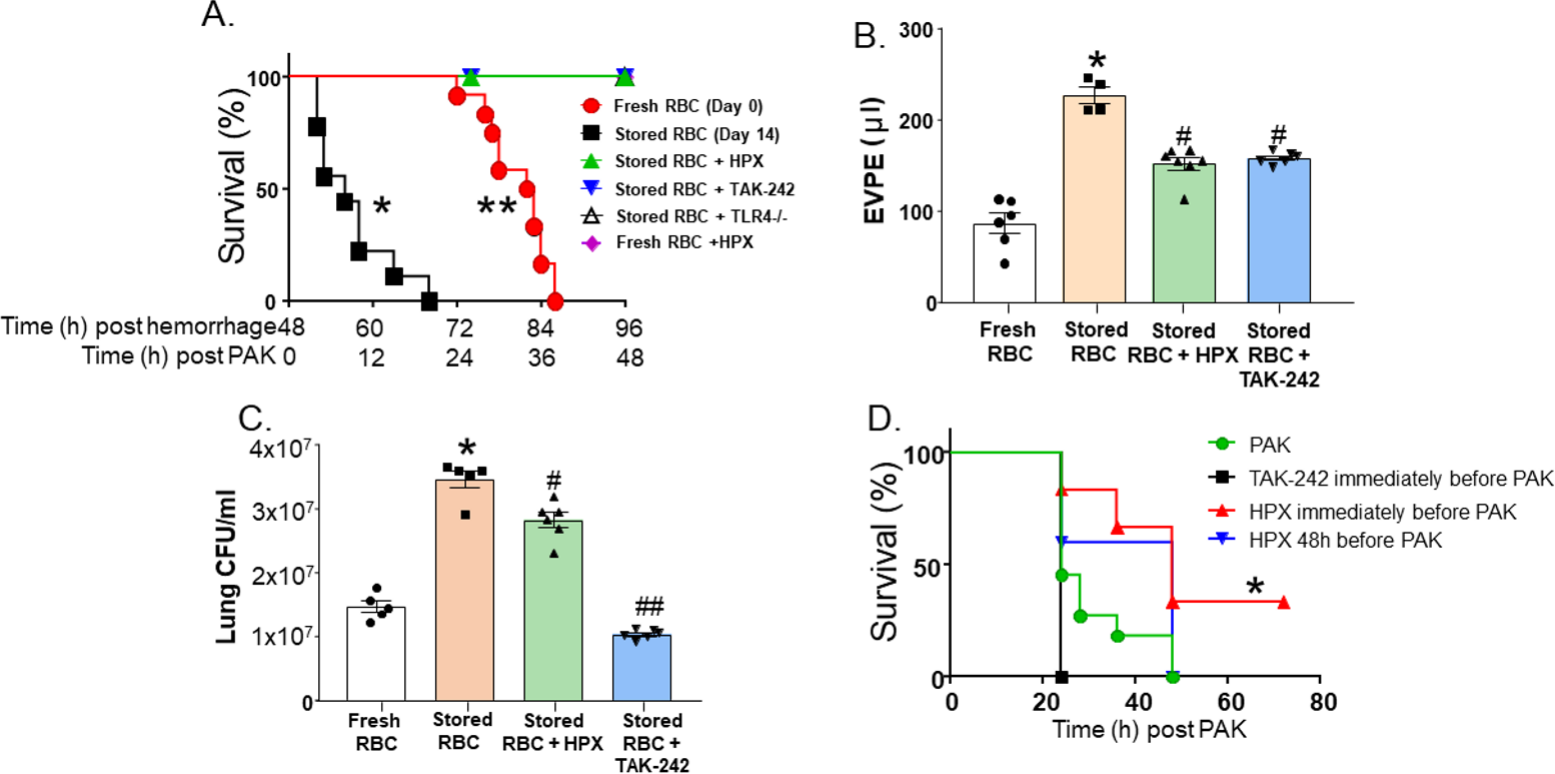
Impairment of lung function secondary to trauma hemorrhage (TH) and resuscitation with stored blood and subsequent pneumonia is heme- and TLR4-dependent. C57BL/6 or *TLR4*^−/−^ mice underwent TH with fresh or stored red blood cell (RBC) resuscitation (with plasma 1:1) and subsequent *P*. *aeruginosa* K-strain (PAK)–induced pneumonia 48 h thereafter. Mice also received vehicle, hemopexin (HPX, 1 mg/kg in 100 μl of PBS) intravenously immediately before resuscitation, or TAK-242 (7.5 mg/kg) intravenously immediately before hemorrhage. (A) Survival after PAK administration. Each symbol represents an individual mouse. Data from fresh (0 storage days, circles, *n =* 12) or stored (14 storage days, squares, *n =* 9) RBCs are the same as presented in Fig 1A and collected and pooled over the entire project period as described in Methods. Triangles indicate stored RBCs + HPX (*n =* 5, *p < 0.001 relative to stored RBCs by log-rank test); inverted triangles indicate stored RBCs + TAK-242 (*n =* 5, *p < 0.001 relative to stored RBCs by log-rank test); open triangles indicate stored RBCs in *TLR4*^−/−^ mice (*n =* 5, *p < 0.001 relative to stored RBCs by log-rank test); diamonds indicate fresh RBCs + HPX (*n =* 4, **p = 0.002 relative to fresh RBCs by log-rank test). (B) Mice underwent TH and resuscitation with either fresh (*n =* 6) or stored RBCs (*n =* 4), stored RBCs and hemopexin (*n =* 7), or stored RBCs and TAK-242 (*n =* 6). Forty-eight hours later, PAK was instilled, and 6 h thereafter mice were sacrificed and extravascular pulmonary equivalents (EVPE) measured. Data are mean ± SEM; each symbol represents an individual mouse. *p < 0.001 relative to fresh RBCs and ^#^*p <* 0.001 relative to stored RBCs by 1-way ANOVA with Tukey post-test. (C) Mice underwent TH and resuscitation with either fresh (*n =* 5) or stored RBCs (*n =* 5), stored RBCs and hemopexin (*n =* 6), or stored RBCs and TAK-242 (*n =* 6). Forty-eight hours later, PAK was instilled, and 6 h thereafter mice were sacrificed and lung colony-forming units (CFU)/ml measured. Data are mean ± SEM; each symbol represents an individual mouse. *p < 0.001 relative to fresh RBCs and ^#^*p =* 0.002 and ^##^*p <* 0.001 relative to stored RBCs by 1-way ANOVA with Tukey post-test. (D) Mice were administered PAK only by airway instillation (no TH or stored RBC resuscitation) (circles, *n =* 11), TAK-242 immediately prior to PAK instillation (squares, *n =* 3), or hemopexin immediately (triangles, *n =* 6) or 48 h (inverted triangles, *n =* 5) prior to PAK instillation, and survival monitored. *p = 0.022 for hemopexin administered immediately before PAK relative to PAK alone by log-rank test.

Mice (*n =* 5) were treated with hemopexin after hemorrhagic shock, but before massive resuscitation with stored RBCs, followed by PAK administration 48 h thereafter. Hemopexin improved post-pneumonia survival in mice that underwent massive resuscitation with stored RBCs (Fig 3A). Furthermore, hemopexin improved *P*. *aeruginosa–*induced pulmonary edema formation (Fig 3B; *n =* 4–7) and lung bacterial CFUs (Fig 3C; *n =* 5–6). These data indicate that the increased severity of bacterial lung injury secondary to massive resuscitation with stored RBCs is heme-dependent. Interestingly, mice transfused with stored RBCs and treated with hemopexin had significantly lower hemoglobin levels compared to mice transfused with stored RBCs. Free heme and NTBI levels tended to be lower at 4 h post-transfusion in hemopexin-treated mice; however, this difference did not reach statistical significance (Fig 2A).

### Role of TLR4 cell signaling in lung bacterial infection and injury secondary to TH and massive resuscitation with stored RBCs

Previous studies have shown that free heme can activate the TLR4 receptor [7,36,44,45]. To determine whether the effect of resuscitation with stored RBCs is TLR4-dependent, we treated TH mice (*n =* 5) with TAK-242, a TLR4 inhibitor, or with its vehicle (intralipid) after trauma (laparotomy) and at the onset of shock. Treatment with TAK-242 improved post-pneumonia survival (Fig 3A). Additionally, TAK-242 improved pulmonary edema formation (Fig 3B) and decreased lung bacterial CFUs (Fig 3C). Finally, to demonstrate that our results were not due to off-target effects of TAK-242, we confirmed our survival results in *TLR4*^−/−^ mice. *TLR4*^−/−^ mice (*n =* 5) that underwent TH with massive resuscitation with stored RBCs had significantly improved survival compared to wild-type mice (Fig 3A). These data demonstrate that TLR4 signaling plays a role in heme-induced exacerbation of lung bacterial infection secondary to TH followed by massive resuscitation with stored RBCs.

### Effects of hemopexin and TLR4 blockade are due to prevention of storage-lesion-dependent toxicity

To test if the protection afforded by anti-TLR4 or anti-heme therapies was due to protection against bacterial pneumonia infection per se, rather than storage lesion plus bacterial pneumonia, mice were infected with PAK in the absence of any trauma, hemorrhage, or resuscitation. Fig 3D shows that mice administered PAK all died within 48 h (*n =* 11). TAK-242, administered immediately prior to PAK instillation, had no significant effect on survival (*n =* 3). Interestingly, hemopexin administered at the time of PAK instillation (*n =* 6) did improve survival, consistent with prior reports showing that heme sequestration prevents sepsis-induced injury [7,58]. However, hemopexin administered 48 h prior to *P*. *aeruginosa* instillation (with no trauma, hemorrhage, or stored RBC resuscitation, *n =* 5), which mimics the protocol for hemopexin administered in the data shown in Fig 3A, had no effect on survival after bacterial infection.

### Heme-induced increases in lung endothelial permeability are TLR4-dependent

**Fig 4:**
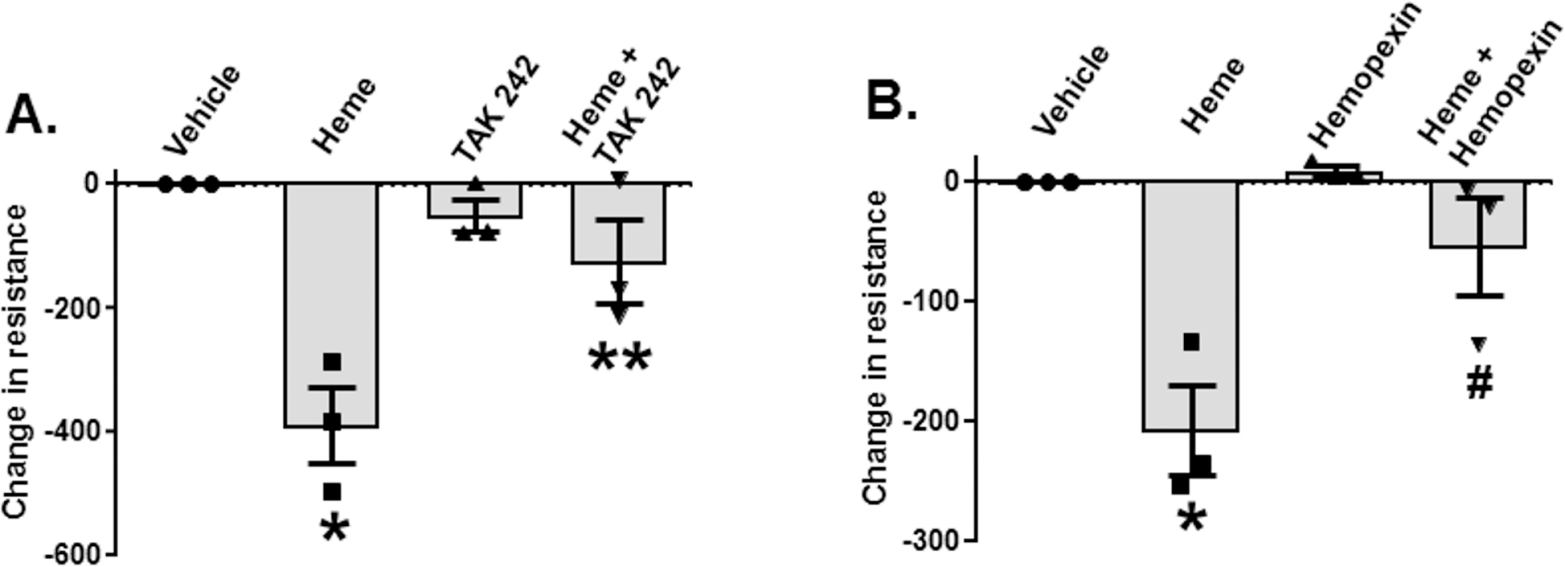
Heme-dependent decreases in endothelial barrier resistance are TLR4-dependent. Rat microvascular endothelial cell (RMVEC) monolayers were seeded in Electric Cell-substrate Impedance Sensing (ECIS) arrays as described in Methods. When monolayers reached the appropriate resistance, monolayers were treated with vehicle or heme (10 μM, *n =* 3) alone or in the presence of TAK-242 (150 μg/ml TAK-242 was added 30 min before administration of heme, *n =* 3) (A) or 10 μM hemopexin (*n =* 3) (B). Resistance was measured every 10 min for 8 h. Changes in resistance after 8 h were calculated and vehicle effects subtracted. Decreases (i.e., negative values) in resistance indicate increases in permeability. Data are mean ± SEM (*n =* 3), with each symbol representing an independent experiment. *p = 0.002 and **p = 0.003 relative to vehicle and ^$^*p =* 0.018 and ^#^*p =* 0.019 relative to heme determined by repeated measures 1-way ANOVA with Tukey post-test.

TH and resuscitation with stored compared to fresh RBCs not only caused increased mortality but also significantly increased the severity of pulmonary edema induced by *P*. *aeruginosa* pneumonia (Fig 1D). Recent studies have shown that heme induces paracellular permeability [59,60]. To test if heme, via TLR4-dependent pathways, induces endothelial permeability, RMVEC monolayers were treated with hemin. Hemin treatment significantly decreased resistance (indicating increased permeability),but this decrease was prevented by TLR4 inhibition using TAK-242 (Fig 4A) and hemopexin (Fig 4B).

### Heme induces exogenous release of HMGB1 that subsequently inhibits phagocytosis of *P*. *aeruginosa* by alveolar macrophages

**Fig 5:**
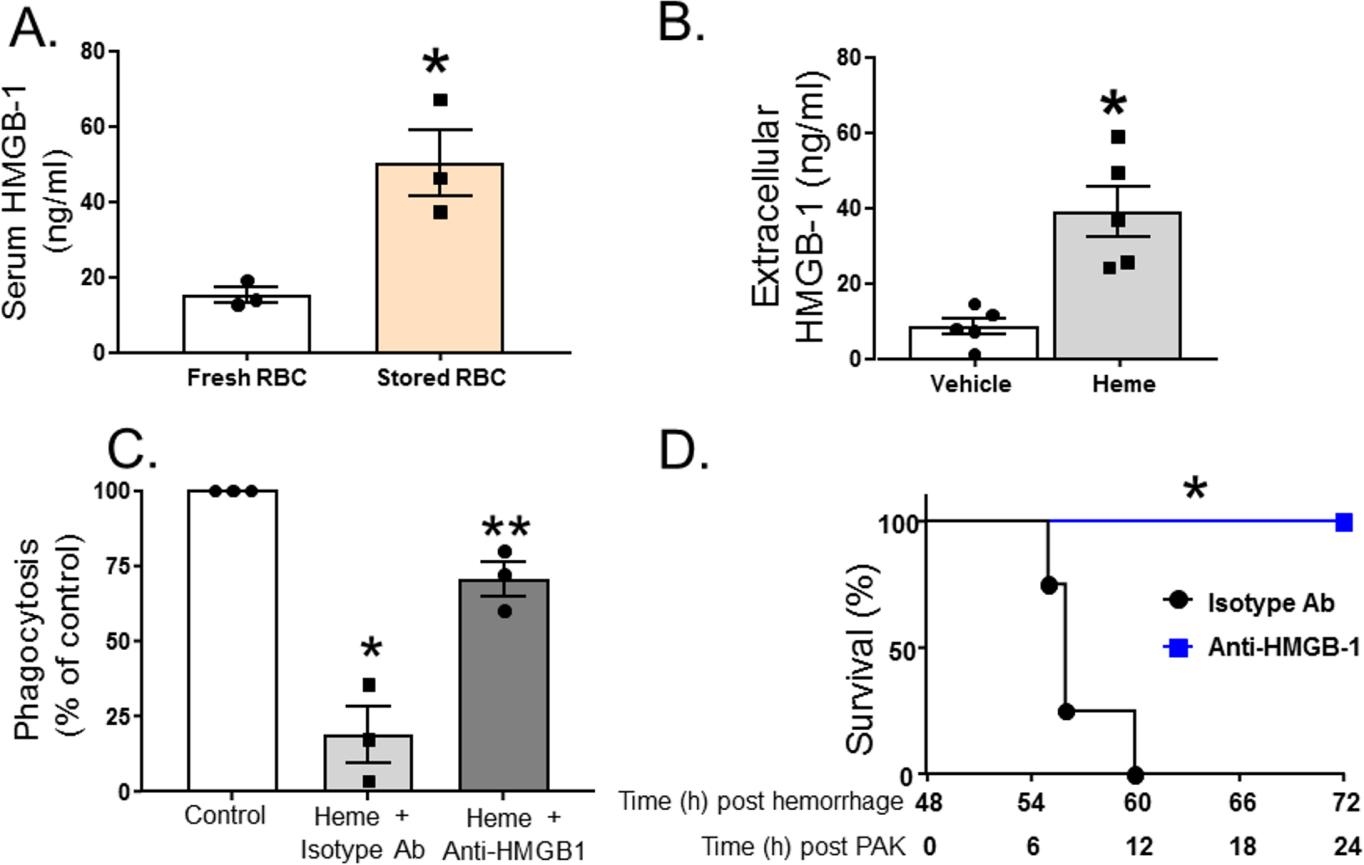
Heme induces exogenous release of HMGB1, which subsequently inhibits phagocytosis of *P*. *aeruginosa* by alveolar macrophages. (A) C57BL/6 wild-type mice underwent trauma hemorrhage (TH) and were randomly resuscitated with either fresh (*n =* 3) or stored (*n =* 3) red blood cells (RBCs) with plasma (1:1). Blood was drawn 6 h after resuscitation, and high mobility group box 1 (HMGB1) levels were measured by ELISA. Data are mean ± SEM; each symbol represents an individual mouse. *p = 0.018 compared to mice resuscitated with fresh blood by unpaired *t* test. (B) Rat microvascular endothelial cell (RMVEC) monolayers were exposed to vehicle (0.01 M NaOH, *n =* 5) or hemin (10 μM, *n =* 5) for 6 h, and supernatants were analyzed by ELISA for extracellular HMGB1. Data show mean ± SEM. *p = 0.003 compared to vehicle by unpaired *t* test. (C) For phagocytosis assays, MH-S cells were pretreated with anti-HMGB1 blocking antibody or isotype control antibody prior to exposure to hemin and *P*. *aeruginosa* K-strain (PAK). Colonies were counted, and phagocytosis expressed as percent of control, with 100 percent being represented by colony number in plates in vehicle-treated cells. Data were collected from 3 independent experiments with 3 replicates in each experiment. Data are mean ± SEM; each symbol represents an individual experimental mean. *p = 0.023 relative to control and **p = 0.045 relative to heme + isotype antibody by 1-way repeated measures ANOVA with Tukey post-test. (D) C57BL/6 wild-type mice underwent TH and resuscitation with stored RBCs with plasma (1:1). Forty-eight hours later, these mice were instilled with PAK, and survival monitored. Before instillation of PAK, mice were injected peritoneally with either anti-HMGB1 blocking antibody (*n =* 6) or isotype control antibody (*n =* 4). *p = 0.001 compared to isotype control by log-rank test.

HMGB1 is a damage-associated molecular pattern molecule that is released by a variety of cells at trauma and tissue injury, and a known mediator of decreased bacterial clearance [61]. We therefore hypothesized that resuscitation with stored RBCs would mediate further increases in extracellular HMGB1 release. Fig 5A shows that extracellular serum levels of HMGB1 were approximately 5-fold higher in mice (*n =* 3) resuscitated with stored RBCs compared to mice resuscitated fresh RBCs. Furthermore, free heme also induced extracellular release of HMGB1 from RMVECs compared to vehicle control (Fig 5B). TH and resuscitation with stored compared to fresh blood not only caused increased *P*. *aeruginosa–*mediated mortality but was also associated with a significantly higher number of bacterial CFUs in the lung. To test if this could be due to an immunosuppression effect of heme, in the next series of experiments, we determined whether exposure to heme inhibited *P*. *aeruginosa* phagocytosis by MH-S cells, a murine alveolar macrophage cell line. The results indicate that exposure to heme caused a 90% inhibition of *P*. *aeruginosa* phagocytosis by MH-S cells (Fig 5C). Furthermore, when MH-S cells were pretreated with an anti-HMGB1 blocking antibody (or isotype control) before exposure to hemin, heme-mediated inhibition of *P*. *aeruginosa* phagocytosis by MH-S cells was attenuated. Finally, mice that underwent TH followed by resuscitation with stored RBCs were injected with anti-HMGB1 blocking antibody (or isotype control) before tracheal instillation of *P*. *aeruginosa* (Fig 5D). As seen, mice who received anti-HMGB1 blocking antibody (*n =* 6) had improved survival after resuscitation with stored RBCs and subsequent *P*. *aeruginosa* pneumonia compared to mice receiving the control antibody (*n =* 4). In summary, these results indicate that heme from resuscitation with stored RBCs increases extracellular release of HMGB1, which inhibits macrophage phagocytosis of *P*. *aeruginosa*, leading to increased mortality.

### Heme levels in patients after TH and resuscitation

**Fig 6:**
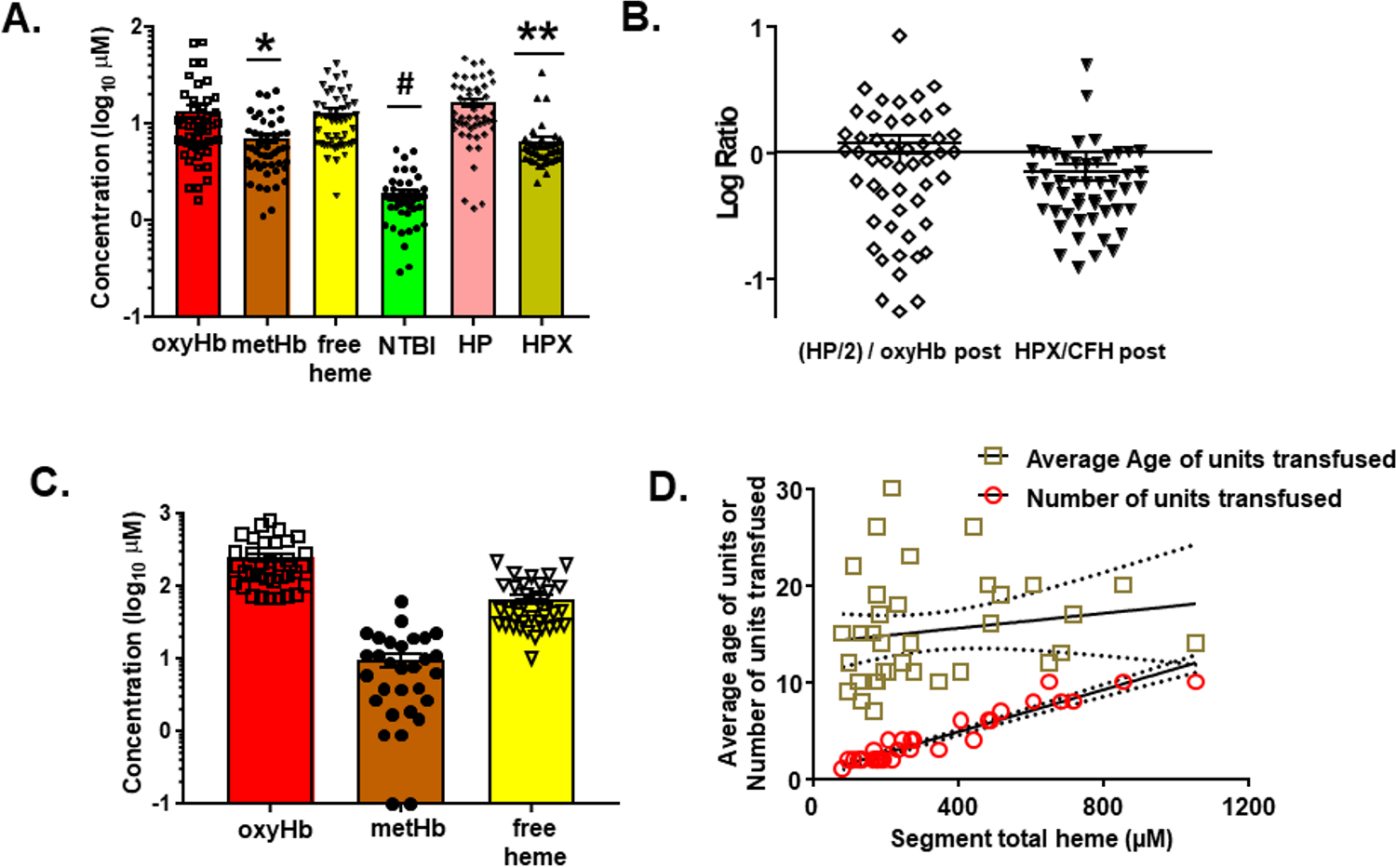
Measurement of hemolysis products and heme-sequestering proteins in transfused units and plasma in trauma hemorrhage (TH) patients. Fifty TH patients requiring blood product resuscitation admitted to the University of Alabama at Birmingham ER between 1 January 2015 and 30 April 2016 were enrolled. (A) Oxyhemoglobin (oxyHb), methemoglobin (metHb), free heme, haptoglobin (HP), and hemopexin (HPX) concentrations in plasma from TH patients 2–3 h after resuscitation. Data are mean ± SEM. *p = 0.002 relative to free heme; #*p <* 0.001 relative to oxyHb, metHb, and free heme; and **p < 0.001 relative to HP and free heme by Kruskal–Wallis test with Dunn’s multiple comparison test. (B) Molar ratios (log transformed) of HP/2:hemoglobin and HPX:heme. The former is presented as HP/2:oxyHb to reflect the fact that haptoglobin binds to hemoglobin dimers. Each data point reflects an individual patient. Data show mean ± SEM. Segments associated with red blood cell (RBC) units transfused and plasma from patients after stable resuscitation were collected. (C) Total oxyHb, metHb, and free heme concentrations in segments of RBC units transfused (*n =* 35). (D) Segment concentrations of total heme were plotted against the average storage age of the transfused RBCs or the number of units transfused. Line represents best fit by linear regression with 95% confidence intervals (dotted lines). Slope was significantly non-zero for number of units (*r*^2^ = 0.91, *p <* 0.001).

To our knowledge, no study has assessed the amount of in vitro products of hemolysis transfused into TH patients. Fifty patients were enrolled in the study. Table 1 shows demographic information. Fig 6A shows the levels of plasma free oxyHb, metHb, heme, NTBI, haptoglobin (HP), and hemopexin (HPX) measured once patients were stably resuscitated (approximately 2–3 h after initiation of resuscitation). OxyHb (median 9.2 μM, IQR 5.8–14.3 μM) and free heme (median 10.9 μM, IQR 6.3–16.4 μM) were similar and both higher than metHb or NTBI level, with NTBI level being approximately 5- to 10-fold lower than oxyHb or CFH. Interestingly, hemoglobin and HP levels were similar, but HPX level was significantly lower than free heme level. Fig 6B plots the ratio of HP/2:hemoglobin and HPX:free heme. The ratio of HP/2:hemoglobin was close to 1 (median 0.87, IQR 0.28–1.50), although variance is noted. For hemopexin:free heme, the ratio was lower (median 0.56, IQR 0.34–0.86) and below 1 (*p <* 0.01 by 1-sample *t* test and comparison to a theoretical mean of 1). Segments attached to blood bags that were transfused into TH patients were also collected at the time of transfusion; data from 35 patients for whom segments from each unit transfused were available were analyzed for supernatant levels of oxyHb, metHb, and free heme. Fig 6C plots the concentrations of transfused oxyHb, metHb, and free heme. Levels varied 20-fold (from micromolar to close to millimolar concentrations). The percentage of oxyHb, metHb, and free heme of total heme for all units transfused was relatively constant, at approximately 75%, 3%, and 22%, respectively, indicating that the total heme concentration transfused was largely dependent on the number of RBC units transfused. Fig 6D confirms this, showing that the total heme (sum of oxyHb, metHb, and free heme) transfused linearly correlated with the number of units transfused. Interestingly, no correlation with the average storage age (15.3 ± 0.9 d, mean ± SEM, *n =* 35) of the RBC units was observed. Fifteen of these 35 patients were massively transfused (defined as an average rate of transfusion of >3 units per hour, assessed until stably resuscitated).

**Table 1:** Trauma hemorrhage patient demographics at admission (*n =* 50 unless otherwise noted).

Characteristic	Value
Sex	36 male, 14 female
Race	25 white, 25 African-American
Age (y) (median, IQR)	33, 25–53
Mechanism of injury	27 blunt, 23 penetrating
Injury Severity Score (median, IQR)	24, 17–34
Diabetes mellitus	45 no, 3 yes, 2 unknown
Admission respiratory failure	22 no, 28 yes
Admission arterial blood pressure (mm Hg) (mean ± SD)	68.9 ± 19.7 (*n =* 47)
Admission Glasgow Coma Score (mean)	11.9 (*n =* 49)
Smoking history	22 never, 23 current, 2 former, 3 unknown
Alcohol use	30 never, 17 current, 3 unknown

## Discussion

Our findings using a mouse model of trauma and hemorrhagic shock with stored blood transfusion and subsequent PAK instillation demonstrate that transfusion with older stored RBCs increases the severity of bacterial pneumonia. Using pharmacological inhibition or genetic ablation approaches in vivo, we further showed that free heme present in stored RBCs, or generated after transfusion with stored RBCs, triggers a TLR4- and HMGB1-dependent process that leads to increased pulmonary microvascular permeability, higher bacterial numbers after PAK instillation in vivo, and inhibition of bacterial clearance by immune cells in vitro. Complementing these observations, we demonstrated that in addition to exposure to increased heme, endogenous hemopexin is limited in resuscitated trauma patients and mouse models of TH.

The safety and efficacy of stored RBCs has received much attention recently, fueled, in large part, by retrospective analyses indicating that transfusion with older stored blood is associated with adverse effects [1–6,8–12]. More recent data from prospective randomized research clinical studies, however, indicate that transfusion with younger versus older RBCs was equivalent in cardiac surgery and critically ill patients [13,14,16]. Notwithstanding limitations with study design, an important question is how generalizable these conclusions are to patients receiving stored RBCs to treat massive bleeding and hemorrhagic shock. We focused on TH patients, in whom associations between stored RBCs and adverse events—characterized by microcirculatory dysfunction, increased critical end organ injury, and inflammation and lung infection—had been shown in multiple retrospective analyses, further supported by preclinical animal model studies [29,36,38,57]. Using a murine model of TH with stored RBC and plasma resuscitation, we found that transfusion with 3 units of leukoreduced mouse RBCs stored for 14 d, which is similar to human RBCs stored for 35–42 d, increases the severity of *P*. *aeruginosa–*induced pulmonary edema and mortality in mice. Importantly, retrospective analyses of clinical data demonstrate associations between the age of blood transfused and increased risk for lung infection [43].

The effect of stored RBCs in increasing the severity of *P*. *aeruginosa* infection was observed up to at least 48 h post-resuscitation. To gain insights into potential mechanisms, we first measured levels of putative mediators formed during RBC storage that may increase lung vascular permeability and/or promote bacterial infection/prevent bacterial clearance. We measured hemoglobin (oxyHb and metHb) and free heme in the stored RBCs, and these mediators and NTBI in mice after transfusion. We used a spectral deconvolution approach to measure heme-containing species, which we recently showed avoids artifacts associated with widely used and commercially available assays that do not distinguish between free heme and hemoglobin [52]. Consistent with our previous reports, levels of hemoglobin and free heme were in the hundreds of micromolar range in stored RBC supernatants [25,52]. These levels led to an increase in circulating free hemoglobin, free heme, and NTBI after resuscitation, indicated by these species being elevated after resuscitating mice with stored RBCs. However, these levels all returned to baseline by 24–48 h, suggesting that some of these mediators may activate cell signaling pathways that lead to increased lung vascular permeability and inhibit the host response to a secondary lung bacterial infection. While our primary hypothesis was that free heme is formed ex vivo by “hemolysis in the bag” before transfusion, mixtures of fresh RBCs with stored supernatants and vice versa produced an intermediate response in terms of survival after secondary pneumonia; mice did not die as quickly as those resuscitated with stored RBCs and tended to die earlier than those resuscitated with fresh RBCs. This suggests that both in-bag hemolysis and heme release during intravascular hemolysis (likely via lysis of older, more fragile RBCs), or possibly extravascular hemolysis after transfusion, are playing a role in sensitizing mice to subsequent PAK-induced injury. These data underscore the need for better understanding of the primary mechanisms and temporal features of free heme release and exposure in the setting of TH with stored RBC transfusion.

There are multiple distinct molecular species that increase post-transfusion that could play a role in stored-RBC-dependent increased susceptibility to *P*. *aeruginosa* toxicity. These include free hemoglobin, which causes microcirculatory dysfunction secondary to nitric oxide scavenging, and NTBI, which may provide substrate for bacterial growth [17,18,32,38]. We focused on free heme for several reasons. Stored-RBC-dependent acute lung and kidney injury is prevented by hemopexin, heme addition promotes endothelial and epithelial injury [36,57,59], and multiple studies have shown heme to be a mediator of acute inflammatory injury to all major organ systems in sickle cell disease and sepsis via multiple overlapping mechanisms including TLR4 activation, oxidative stress, immune cell dysfunction, and suppression of phagocytosis [44–47,58,62,63]. Our data demonstrate that HPX completely reversed *P*. *aeruginosa*–induced mortality in mice transfused with stored RBCs, supporting a central role for free heme in increased infection risk in this pathological setting. Our studies do not exclude a possible role for hemoglobin or NTBI in exacerbated injury after *P*. *aeruginosa* instillation. Assessing the role of these mediators requires further studies, using targeted approaches such as assessing the effects of haptoglobin (to scavenge free hemoglobin) or apo-transferrin (to chelate NTBI) for example. Further underscoring the need for additional studies were data showing that HPX significantly decreased free hemoglobin levels in mice transfused with stored RBCs (Fig 2). Hemopexin binds free heme with a high affinity, and thus it was surprising that CFH levels were not significantly decreased in HPX-treated mice. This likely reflects limitations of sampling at a single time point coupled with inherent variation in this marker. More surprising was the observation that HPX significantly decreased free hemoglobin levels. Hemopexin does not bind hemoglobin, and assuming that free heme is derived from hemoglobin in this model, these data suggest that free heme mediates, in part, red cell hemolysis and/or stimulates endogenous clearance of hemoglobin. We speculate that the pro-oxidative properties of free heme contribute to ongoing hemolysis, providing a feed-forward process resulting in more hemoglobin and heme release. This could also explain why the HPX therapy is so potent, resulting in almost complete protection in this and other models of hemolysis, where the toxicity is mediated not just by free heme, but also by hemoglobin. Finally, we note that, using survival after PAK instillation as an end point, both HPX and TLR4 inhibition or depletion showed protective effects against stored RBC toxicity that went beyond the protective effects of using fresh RBCs. All mice still died after PAK in the fresh RBC group by 40 h, but in HPX-treated and TLR4-inhibited mice resuscitated with stored RBCs, no deaths were observed up to 48 h after PAK. Interestingly, HPX also improved survival after PAK when it was administered 48 h after TH and fresh RBC resuscitation. This suggests that the toxicity that occurs with fresh RBCs is also heme-dependent. However, no differences in survival were observed between PAK alone and PAK after TH with fresh RBCs (compare survival curves in Fig 3A and 3D, suggesting no gain of toxic function associated with fresh RBCs). That said, HPX administered 48 h prior to PAK, in the absence of TH and RBC resuscitation, had no effect on survival after PAK (see below and Fig 3D). Thus, we conclude that heme is a mediator of PAK infection with or without fresh/stored RBC transfusion after TH, but with TH and RBC transfusion, the primary source of heme is RBC-dependent, and the severity of injury is greater with stored RBCs. Moreover, the effects of heme occur early as treatment with HPX or TLR4 inhibitors is sufficient to prevent subsequent effects of PAK instillation, underscoring the critical effects of heme exposure in altering the course of subsequent injury after TH and shock.

It is possible that the improved survival mediated by TLR4 inhibition or HPX therapy was due to protection against bacterial pneumonia rather than against storage lesion plus bacterial pneumonia. However, TAK-242 had no effect on survival after PAK instillation alone (in the absence of trauma, hemorrhage, and stored RBC resuscitation), suggesting that the protective effect of TLR4 inhibition on PAK-induced mortality after TH and stored RBC resuscitation is due to mitigation of storage-lesion-dependent processes. These data are consistent with published reports showing no differences in PAK pneumonia in wild-type versus TLR4-deficient mice [64]. While HPX administered at the time of PAK instillation did show protective effects, when administered 48 h prior to PAK instillation—mimicking the protocol used for our study of TH with stored RBC resuscitation—no protection was observed. Therefore, we conclude that while HPX can prevent bacterial sepsis, reproducing prior reports [7,58], the protective effects of HPX observed in the presence of TH and stored RBC resuscitation are largely due to protection against storage-lesion-derived heme-mediated immunosuppression.

**Fig 7:**
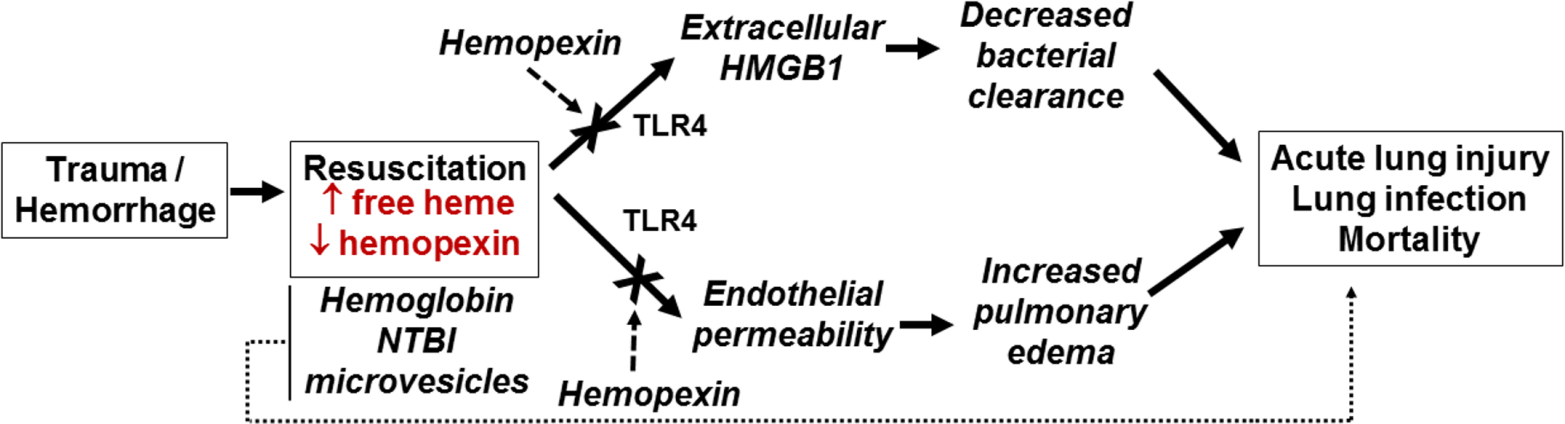
Proposed mechanisms for free-heme-dependent potentiation of lung infection after trauma hemorrhage. We hypothesize that when resuscitation with stored red blood cells (RBCs) leads to increased circulating heme that is in excess of hemopexin, 2 parallel pathways are activated involving (i) release of HMGB1 and subsequent decreased immune-cell-dependent bacterial clearance and (ii) increased endothelial permeability. These combine to promote acute lung injury and increase sensitivity to lung infection. We also underscore the potential that heme-dependent effects synergize with other components of stored RBCs, including hemoglobin, non-transferrin-bound iron (NTBI), and microvesicles, to mediate adverse outcomes.

We demonstrated that free heme promotes mortality secondary to *P*. *aeruginosa* infection, and propose 2 parallel mechanisms underlying this effect (Fig 7). These involve induction of endothelial permeability and inhibition of bacterial clearance mediated by TLR4 and HMGB1, respectively. First, while the role of TLR4 in TH has been widely studied, the potential for transfused heme as a TLR4 ligand in this setting has not been explored in detail. Heme can bind TLR4 and activate downstream signaling. This pathway appears to be pivotal as *P*. *aeruginosa–*induced mortality after stored RBC transfusion was completely reversed by TLR4 blockade during TH or by genetic ablation of *TLR4*. We also demonstrated in vitro that endothelial TLR4 is a key mediator in heme-induced permeability. However, further studies with tissue-selective *TLR4* knockout animals are needed to test the relative role of endothelial TLR4 in mediating decreased lung bacterial clearance and alveolar macrophage phagocytosis after TH and stored RBC resuscitation.

Extracellular HMGB1 is a damage-associated molecular pattern that elicits multiple effects that promote end organ damage. These include increasing the severity of lung injury subsequent to bacterial infection [65,66], promoting neutrophil extracellular trap formation [67], inhibiting macrophage efferocytosis of apoptotic neutrophils [68], and decreasing effective bacterial killing via the inhibition of neutrophil NADPH oxidase [61]. We have shown that plasma HMGB1 is higher in transfused trauma patients compared to those who were not transfused [69]. We extend these observations in a mouse TH model, demonstrating that stored RBC resuscitation results in higher plasma HMGB1 compared to fresh RBC resuscitation. A recent study indicated that in non-leukoreduced RBC preparations, storage resulted in increased extracellular HMGB1 that inhibited macrophage-dependent phagocytosis [70]. However, HMGB1 levels in supernatants are dramatically decreased in leukoreduced stored RBCs, and since we used the latter, we suggest stored RBCs are unlikely to directly account for the increased plasma levels of HMGB1 observed after stored RBC resuscitation. A more likely scenario in our studies is that HMGB1 was released via cell death induced by stored RBC resuscitation. Indeed, the latter has been shown to induce lung endothelial cell necroptosis, leading to extracellular release of HMGB1 [71], a result we recapitulated using free heme alone. More important, perhaps, is the fact that anti-HMGB1 blocking antibody significantly improved survival after stored RBC resuscitation and subsequent *P*. *aeruginosa* lung infection in vivo, suggesting that extracellular HMGB1 plays a key role in mediating decreased lung bacterial clearance after resuscitation with stored RBCs. This conclusion was further supported by the finding that anti-HMGB1 blocking antibody restored the ability of macrophages to phagocytose in the presence of free heme. The exact mechanism linking heme and HMGB1 to inhibition of macrophage-dependent phagocytosis remains to be elucidated. Recent studies demonstrated a mechanism involving heme-dependent disruption of actin cytoskeleton dynamics [58], but whether this involves TLR4 or HMGB1 is unclear. Taken altogether, these data support the model proposed in Fig 7, in which heme derived from stored RBCs promotes both endothelial permeability and immunosuppression, defined here as suppressed bacterial clearance. The 2 pathways then converge to increase the severity of lung infection caused by *P*. *aeruginosa*. Additional studies are required to test whether this model is applicable to other bacterial strains and species and to test the role of pro-bacterial growth factors, including iron, that are also increased after stored RBC transfusions. In this context, we emphasize that it is unlikely that a single mediator or mechanism is responsible for stored-RBC-dependent increase in lung injury and infection, and that additive or synergistic interactions between hemoglobin, free heme, NTBI, and microvesicles are essential in adverse outcomes. Additionally, it is important to remember that these species are not independent factors but intimately related, as free heme is hypothesized to be released from free hemoglobin. Indeed, it is possible that some of the protective effects of HP and HPX derive from preventing NTBI formation from hemoglobin and heme degradation, as discussed above. Moreover, mechanistically, additive or synergistic effects between decreased nitric oxide bioavailability, oxidative stress, inflammation, and immunosuppression are likely. Our studies were not designed to study the injurious dose effect of stored blood. Indeed, in our experimental model, mice were resuscitated with a large amount of stored blood that mimics massive blood transfusion in humans to treat the hemorrhagic shock. However, we were careful not to overload the mice with larger than necessary doses of stored blood and plasma that could induce a transfusion-associated circulatory overload. Thus, while our mouse model of TH represents well the injury that occurs secondary to stored RBC transfusion, it does not provide insight into the injurious dose effect of RBCs that may be seen in trauma patients who receive only a few units of stored RBCs. Future studies testing different RBC doses or equal volume resuscitations comprising different numbers of fresh and stored units are warranted to assess dose-dependent effects.

Although the role of heme and other stored RBC components has been investigated in animal models of stored RBC toxicity, very little is known about these mediators in human TH with stored RBC resuscitation. We thus measured the total amount of hemoglobin and free heme administered to TH patients, and while the total amount varied depending on the number of units transfused, hemoglobin levels were on average approximately 4 times higher than free heme levels. These data show that relatively high concentrations of hemoglobin and free heme are transfused, reaching millimolar concentrations in some cases. Limitations of these measurements include the inability to account for storage age, the varying number of units transfused per patient, and intrinsic donor to donor heterogeneity in storage-dependent hemolysis. Another limitation of these measures is that we sampled segments attached to the bags transfused due to constraints limiting the ability to sample the bags directly. Previous studies have shown that segment levels of free hemoglobin are higher than those of their paired bags, whereas the opposite relationship has been reported for free heme [24,25], suggesting that the relative amount of free heme administered is in fact be higher than shown.

We also measured plasma levels of free hemoglobin, free heme, NTBI, HP, and HPX in TH patients after resuscitation and hemodynamic stability. Plasma free hemoglobin levels were comparable to free heme levels, and both levels were higher than NTBI levels. A large variance was observed, but the majority of patients had levels of free heme above 5 μM, concentrations which are in the range that induce cell permeability and injury in experimental studies. The levels of hemolysis products transfused assessed by segment sampling also varied; however, this variation was not dependent on the average age of the units, but the number of units transfused. This underscores other datasets showing that age per se is not necessarily a good predictor of stored RBC quality, and that volume of RBCs transfused should be considered in evaluating stored RBC toxicities. There are several possible confounders and limitations with these analyses, including the fact that we only assessed a single time point post-transfusion and there is likely some free heme generated post-transfusion by intravascular hemolysis in addition to any administered by the transfusion itself. While the absolute concentration of these mediators is important, the relative concentrations of hemoglobin and free heme, compared to HP and HPX, respectively, are likely of greater clinical significance; HP and HPX are the endogenous primary defense mechanisms protecting against hemolysis-dependent injury. We showed that HP levels were higher than HPX levels, with the HP/2:oxyHb ratio being on average close to 1. In contrast, the HPX:free heme ratio was below 1, suggesting that endogenous protection provided by circulating levels of HPX against free heme is limited, at least over the early time period post-resuscitation. It should, however, be pointed out that these measurements were limited to 1 time point at the end of hemostatic resuscitation and that the data are not powered to test whether there are associations between free hemoglobin, free heme, NTBI, haptoglobin, hemopexin, and clinical outcomes, although the relative assessment of hemoglobin and HP and of free heme and HPX may be of clinical importance. It is also interesting to note that transfusion of plasma is an important component of damage control resuscitation after severe trauma and may provide a significant amount of HP and HPX to protect against products of stored RBC hemolysis.

Study limitations include those noted above regarding the need for a more comprehensive, time-dependent assessment of how products of hemolysis change in both the mouse model used and patients undergoing resuscitation after trauma and hemorrhagic shock. Also as noted above, our studies do not exclude roles for hemoglobin and NTBI in contributing to the increased bacterial burden in the lung mediated by stored RBCs, and we acknowledge the need to measure tissue iron levels and other forms of iron (e.g., transferrin-bound iron) that may contribute to the pathogenesis of bacterial pneumonia.

In summary, using a murine model of trauma and hemorrhagic shock, large volume resuscitation with stored blood, mimicking massive blood transfusion, overcomes endogenous antioxidant defenses and promotes lung bacterial infection. We propose that heme present in stored blood induces lung immunosuppression, indicated by inhibition of bacterial clearance in vitro, resulting in increased mortality from subsequent *P*. *aeruginosa* pneumonia that occurs in a hemopexin/heme-, TLR4-, and HMGB1-dependent manner. We recognize that many challenges and questions remain and view our data as hypothesis generating. Clinically, our findings underscore the need to establish whether the storage age of transfused RBCs correlates with increasing levels of free heme after transfusion, and whether low ratios of HPX to free heme and/or HP to free hemoglobin are associated with adverse outcomes. If demonstrated, this could provide markers for identifying TH patients at higher risks for adverse outcomes. Collectively, our studies underscore the need for prospective clinical studies to test whether the storage age and amount (dose) of stored RBCs transfused is associated with adverse events in severely traumatized patients with hemorrhagic shock that include, but are not limited to, an increased risk for nosocomial infections and end organ injury.

## Supporting information

S1 Data Raw data presented in Figs 1–6. (XLSX)

## Abbreviations

CFH: cell-free heme
CFU: colony-forming unit
EVPE: extravascular pulmonary equivalents
metHb: methemoglobin
NTA: nitrilotriacetic acid
NTBI: non-transferrin-bound iron
oxyHb: oxyhemoglobin
PAK: *Pseudomonas aeruginosa* K-strain
RBC: red blood cell
RMVEC: rat microvascular endothelial cell
TH: trauma hemorrhage
UAB: University of Alabama at Birmingham
