# Correction: Role of heme in lung bacterial infection after trauma hemorrhage and stored red blood cell transfusion: A preclinical experimental study

**DOI:** 10.1371/journal.pmed.1002991

**Published:** 2019-11-13

**Authors:** Brant M. Wagener, Parker J. Hu, Joo-Yeun Oh, Cilina A. Evans, Jillian R. Richter, Jaideep Honavar, Angela P. Brandon, Judy Creighton, Shannon W. Stephens, Charity Morgan, Randal O. Dull, Marisa B. Marques, Jeffrey D. Kerby, Jean-Francois Pittet, Rakesh P. Patel

There is an error in Panel B of Fig 6 and the associated caption. The authors have provided a corrected Fig 6 and caption here.

**Fig 6:**
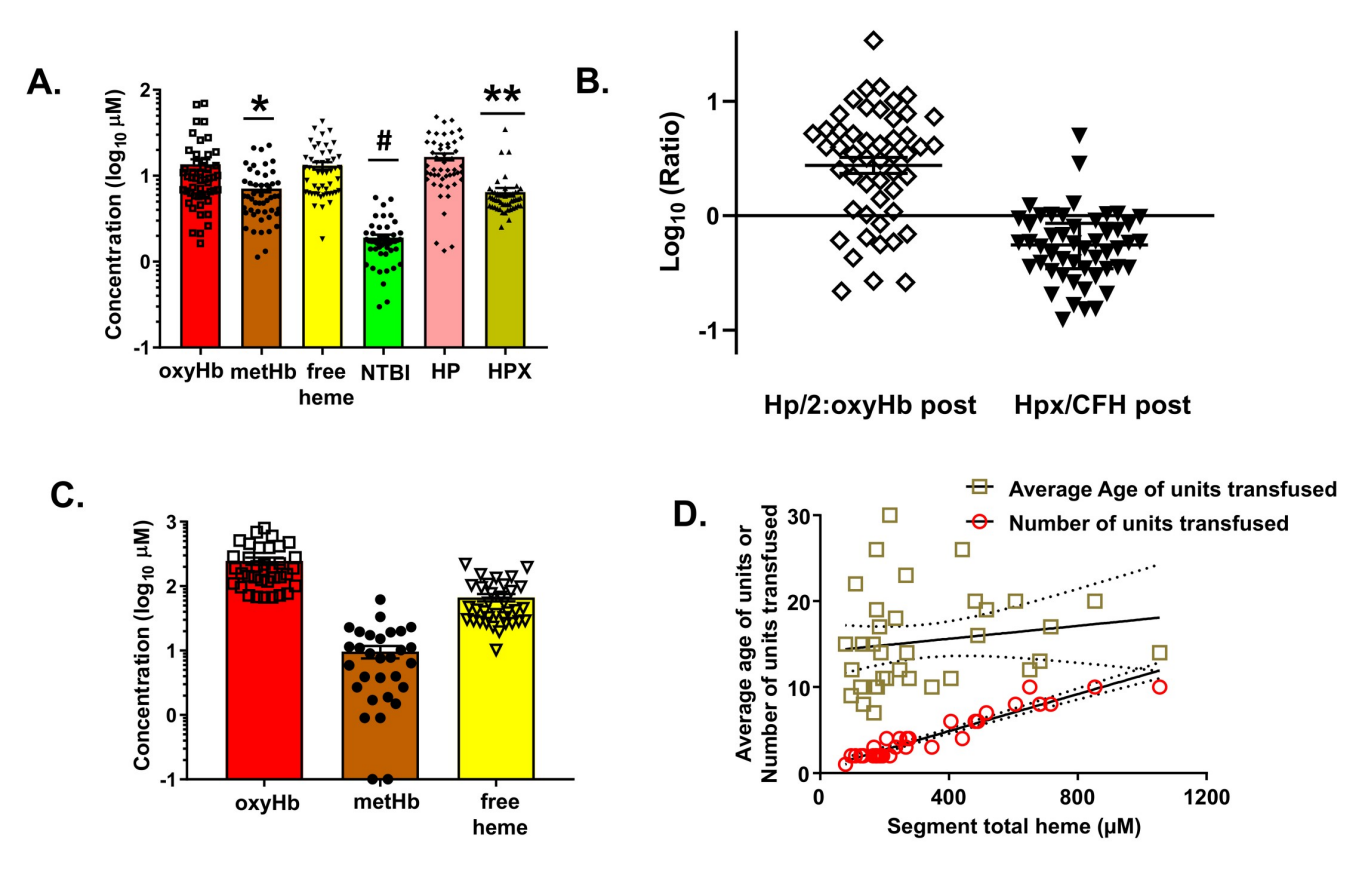
Measurement of hemolysis products and heme-sequestering proteins in transfused units and plasma in TH patients. Fifty TH patients requiring blood product resuscitation admitted to UAB ER between Jan 2015 and Apr 2016 were enrolled. Panel A: oxyHb, metHb, free heme, haptoglobin (Hp) and hemopexin (Hpx) concentrations in plasma from TH patients 2-3h after resuscitation. Data are mean ± SEM. *p = 0.0018 relative to free heme, #p<0.001 relative to oxyHb, metHb and free heme, **p<0.001 relative to Hp and free heme by Kruskal-Wallis with Dunn’s multiple comparison test. Panel B: Molar ratios (log transformed) of Hp:hemoglobin dimer and Hpx:heme. The former is presented as Hp/hemoglobin dimer to reflect the fact that one haptoglobin binds to one hemoglobin dimer. Each data point reflects an individual patient. Data show mean ± SEM. Segments associated with RBC units transfused and plasma from patients after stable resuscitation was collected. Panel C: total oxyHb, metHb and free heme concentrations in segments of RBC units transfused (n = 35). Panel D: Segment concentrations of total heme were plotted against the average age of the transfused RBC or the number of units transfused. Line represents best fit by linear regression with 95% confidence intervals. Slope was significantly non-zero for number of units (r2 = 0.91, p<0.001).

In the Results, in the subsection “Heme levels in patients after TH and resuscitation”, there is an error in the fifth sentence of the first paragraph. The correct sentence is: Fig 6B plots the ratio of Hp/2:hemoglobin and Hpx:free heme. The ratio of Hp/2:hemoglobin was greater than 1 (median = 3.5, IQR 1.1–6.0, p<0.01 by one sample t-test and comparison to a theoretical mean of one), although variance is noted. For hemopexin:free heme, this ratio was lower (median = 0.56, IQR 0.34–0.86) and below one (p<0.01 by one sample t-test and comparison to a theoretical mean of one).

In the Discussion, there is an error in the seventh sentence of the eighth paragraph. The correct sentence is: While the absolute concentration of these mediators is important, the relative concentrations of hemoglobin and free heme, compared to Hp and Hpx respectively is likely of greater clinical significance; Hp and Hpx are the endogenous primary defense mechanisms protecting against hemolysis-dependent injury. We show that Hp levels were higher than Hpx, with the Hp:hemoglobin dimer ratio being greater than 1.

There is an error in the tab “Fig 6A–6B” of the S1 Data file. The authors have provided a corrected S1 Data file here.

## Supporting information

S1 Data Raw data presented in Figs 1–6. (XLSX)
